# Engineering MIL-53(Al) MOF with Carbon Dots for Synergistic Photocatalysis Applications in Organic Dye Degradation

**DOI:** 10.1021/acsomega.4c11113

**Published:** 2025-04-30

**Authors:** Tanzeel Ul Rehman, Simonpietro Agnello, Franco Mario Gelardi, Antonino Madonia, Alice Sciortino, Martina Maria Calvino, Giuseppe Lazzara, Gianluca Minervini, Annamaria Panniello, Gianpiero Buscarino, Marco Cannas

**Affiliations:** † Dipartimento di Fisica e Chimica Emilio Segrè, Università degli Studi di Palermo, Palermo 90123, Italy; ‡ Institute for Chemical and Physical Processes Bari Division, Italian National Research Council, Bari 70126, Italy

## Abstract

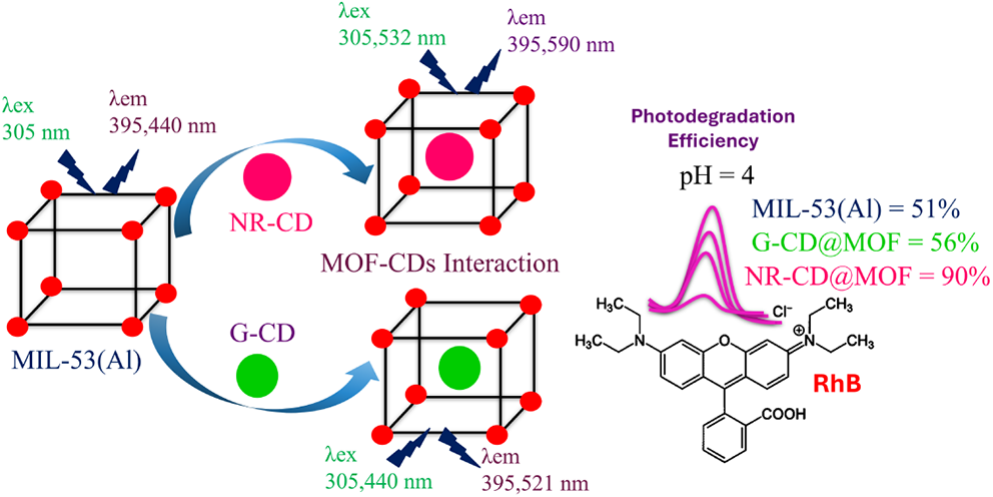

This study investigates the integration of green- and red-emitting carbon dots into the MIL-53(Al) MOF, exploring the structural and optical properties and their impacts on photocatalytic capabilities regarding the photodegradation of the organic dye Rhodamine B. Both types of carbon dots significantly enhance the degradation efficiency, particularly the composites with neutral-red carbon dots at different pH levels. Furthermore, UV–vis absorption and time-resolved photoluminescence spectroscopies provide evidence of electronic structure modifications, such as the enhanced charge separation and the observation of new emission bands related to carbon dots. These findings agree with the structural and optical changes induced by carbon dots integration into the MIL-53(Al) MOF. In general, this research work elucidates the transformative potential of carbon dots and MOF-based composites for advanced material applications, including catalysis and environmental remediation.

## 1 Introduction

The growing environmental pollution and energy crisis impose significant challenges to modern society, requiring the development of innovative and sustainable solutions.^1^ In particular, the increasing contamination of water bodies by organic dyes from industrial effluents has raised significant environmental and health concerns.^2^ A significant example is Rhodamine B (RhB), a widely used dye in the textile, paper, and food industries that is a persistent pollutant in aquatic ecosystems owing to its high stability and resistance to biodegradation.^3,4^ Among various approaches for dye removal, conventional methods, such as coagulation, adsorption, and biological treatment, often fall short in achieving complete decolorization and mineralization.^5,6^ Therefore, photocatalysis is increasingly regarded as an environmentally friendly and facile method to degrade pollutants and produce valuable chemicals, and the development of efficient and sustainable photocatalytic materials is of paramount importance.^7−10^

To this end, in this study, we focus on the photocatalytic capabilities of extremely versatile materials: metal–organic frameworks (MOFs). In fact, the aromatic nature of MOF ligands allows for charge-separated states upon irradiation, enhancing their light-harvesting properties and making them effective photocatalysts.^11−13^ MOF-based photocatalysts outperform the conventional ones owing to their optimal pore dimensions, tunable molecular frameworks, and ease of synthesis, which result in high crystallinity and diverse morphologies.^11,13,14^ The porous nature of MOFs, with open secondary building units (SBUs), facilitates the adsorption and decomposition of organic dyes.^15^ Moreover, postsynthetic modification can further enhance their efficiency in the photochemical decomposition of organic dyes, which are significant contaminants in water bodies.^16,17^

Among various microporous MOFs, MIL-53(Al) has attracted substantial attention due to its unique breathing behavior, variable pore size (from 0.65 to 1.74 nm),^18^ tunable luminescence, and robust thermal and chemical stability.^19^ However, the photocatalytic performance of pristine MIL-53(Al) is often limited by its relatively wide band gap, rapid recombination of photogenerated electron–hole pairs, and suboptimal light absorption.^20,21^ To address the limitations of current photocatalytic materials, this study examines the synthesis of MIL-53(Al) composites with carbon dots (CDs). CDs are emerging nanomaterials with sizes typically less than 10 nm, possessing exceptional optical properties, excellent water dispersibility, high conductivity, high quantum yield (QY), cost-effectiveness, excellent electron transfer capabilities, and good biocompatibility, making them ideal for photocatalytic applications.^22,23^ Therefore, integrating CDs with MOFs is expected to considerably improve the photocatalytic performance of these materials by enhancing charge separation and extending the light absorption spectrum in the visible region.^23,24^

In this study, we synthesized and characterized MIL-53(Al) composites with two innovative types of CDs: green-emitting carbon dots (G-CDs) and neutral red-derived carbon dots (NR-CDs). The rationale behind the selection of these CDs was motivated by their distinct optical absorption and emission properties, which enable a comparative analysis of their effects on the photocatalytic performance of CDs–MOF composites. G-CDs are known for their broad absorption that extends from UV to blue and a green emission, while NR-CDs absorb in the yellow-green spectral region and emit in the red one. Both CDs are capable of strong interactions with the local environment, especially with metal ions through charge transfer processes. Then, these properties are hypothesized to be relevant to the performance of MOF composites toward the photocatalytic degradation of pollutants by extending their light absorption range and improving charge separation. In our experiments, the photocatalytic activity of the composite materials is evaluated through the degradation of RhB dye under UV–visible light. The results, as shown below highlight a remarkable enhancement in photocatalytic efficiency with the composite materials under different pH levels, highlighting the synergistic effect of the CDs and the MIL-53(Al) framework. This research contributes to the design of high-performance photocatalysts through MOF-CDs hybridization and provides effective strategies for environmental remediation. The integration of CDs into MOFs represents a versatile and promising approach to overcoming current limitations in photocatalytic applications, paving the way for future advancements in this field.

## 2 Materials and Methods

### 2.1 Synthesis

**Figure 1:**
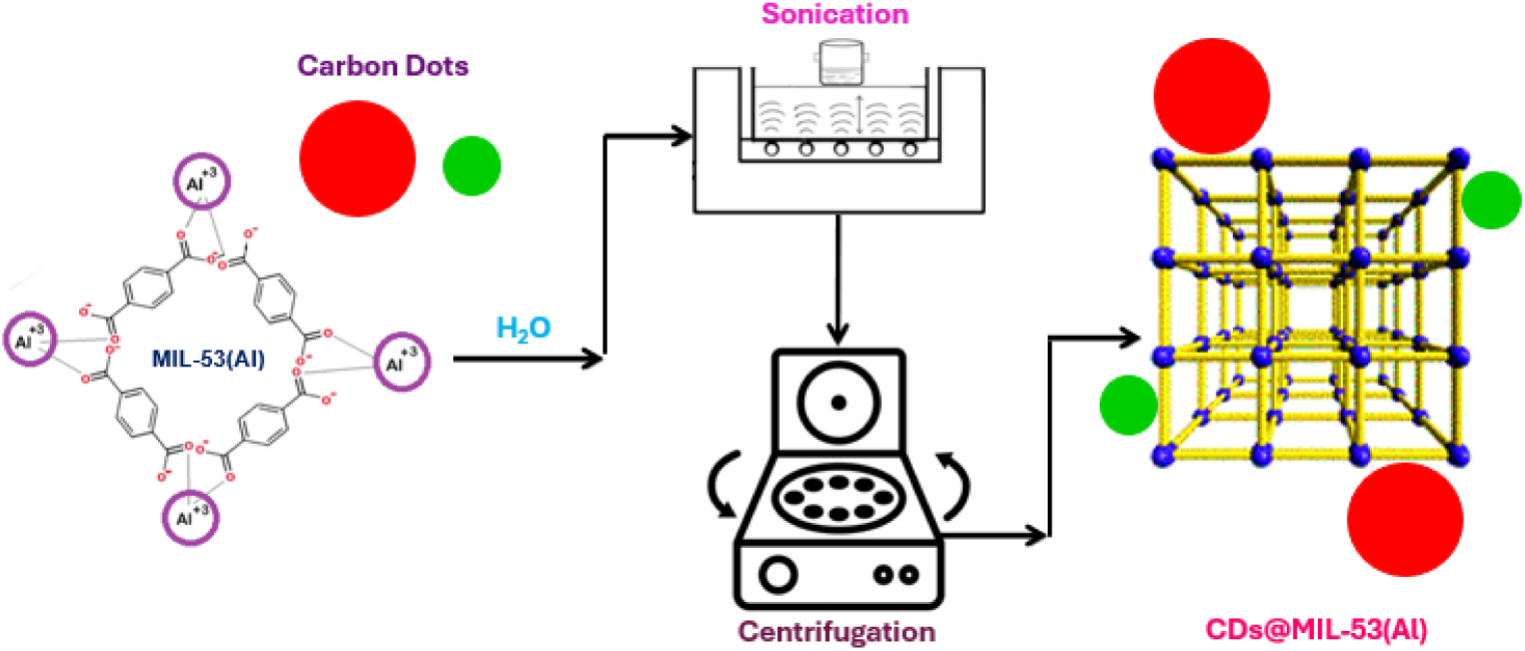
Schematic representation of the synthesis route of the CDs@MOF composites. NR-CDs and G-CDs have sizes of 2 and 1 nm, respectively, which are scaled according to the pore sizes of MOF.

MIL-53(Al) MOF employed in this study was purchased from Sigma–Aldrich (Sigma–Aldrich S.r.l., Milan, Italy) and was used without any further modifications. G-CDs were synthesized by a solvothermal synthesis approach using ammonia and citric acid as precursors through a synthetic protocol already reported in a previous study.^25^ In already conducted atomic force microscopy (AFM) and transmission electron microscopy (TEM) analyses, the diameter of these G-CDs was found to be distributed from 1 to 6 nm, with an average value of ∼3 nm.^26^ Similarly, NR-CDs were also synthesized by a solvothermal synthesis route utilizing neutral red dye and ethylene glycol as precursors in an autoclave under controlled temperature and pressure conditions inside the reaction chamber, as explained in previously reported studies.^27^ Moreover, TEM-based analysis evidenced that the diameter of NR-CDs is distributed from 2 to 10 nm, with an average value of ∼5 nm.^27^ As depicted in the schematic diagram of Figure 1, the composites were prepared by dissolving 300 mg of MIL-53(Al) and 20 mg of CD powders in 3 mL of ultrapure water, followed by sonication of the dispersion for at least 40 min. After the sonication process, a partial degradation of MIL-53(Al) was observed, with only 40 to 60% of the initial product surviving this step as a precipitate. Thereafter, centrifugation was performed at 10 000 rpm for 10 min, collecting the composite in the precipitate and repeating the same procedure three times by washing the samples at each step with distilled water. At the end of the procedure, the sample powders were collected and dried in an oven at 50 °C. Considering the observed MIL-53(Al) mass loss and assuming total incorporation, the CD mass approximately represents between 11% and 15% of the compound.

### 2.2 Characterization Techniques

Structural analysis of pristine MIL-53(Al) MOF, CDs, and their corresponding CDs@MOF composites was performed using the powder X-ray diffraction (PXRD) technique, equipped with a Rigaku Miniflex diffractometer having a Cu Kα source (λ = 1.541 Å). Diffraction data for all the samples were collected at a 0.01° step size and 1°/min, with 2θ angles ranging from 3° to 70°, respectively. To assess the thermal stability of pristine materials and CDs@MOF composites, thermal gravimetric analysis (TGA) and differential thermal analysis (DTA) were performed. The experiments were conducted on a TGA 550 (Discovery Series – TA Instruments). Each sample was heated in a platinum pan from 25 to 750 °C at a scanning rate of 20 °C/min. This thermal treatment was done under inert atmosphere, with 60 and 40 cm^3^ min^–1^ nitrogen flows for the sample and the balance, respectively.

Time-resolved photoluminescence (TRPL) spectroscopy was performed to analyze the emission bands and the kinetic decay of the pristine CDs, MIL-53(Al), and their corresponding composite samples, both in solid-state as well as the aqueous dispersion. The excitation source comprised an optical parametric oscillator (VIBRANT OPOTEK) pumped by the third harmonic (*E* = 3.49 eV) of a Nd:YAG laser with a pulse width of 5 ns and a repetition rate of 10 Hz, respectively. The light emission was analyzed by a monochromator equipped with a 150 lines/mm grating having a 300 nm blaze and then acquired by a delay-generator-driven intensified CCD camera (PIMAX Princeton Instruments). We measured the emission spectra with a 5 nm bandwidth and set the acquisition time window (TW) and delay (TD) in relation to the arrival of the laser pulses on the samples. We observed that the laser pulse has a time decay of approximately 1 ns; therefore, this represents the lower limit for measuring the luminescence lifetime. Finally, the optical properties of the pristine as well as the CDs@MOF composite materials were investigated by UV–vis absorption spectroscopy using a xenon lamp light source connected to a fiber-optic spectrophotometer (Avantes, Apeldoorn, The Netherlands) and steady-state luminescence using an Agilent Cary Eclipse spectrofluorometer mounting a xenon lamp excitation source.

### 2.3 Photocatalysis Experimental Setup

The photocatalytic efficiency of pristine MIL-53(Al) MOF and the CDs@MIL-53(Al) composites was analyzed using RhB as a representative common industrial pollutant. A specific quantity of photocatalyst powder (0.5 mg) was homogeneously dispersed in 3 mL of an RhB aqueous solution. Prior to light exposure, the solution was stirred for 40 min in the dark to achieve the adsorption–desorption equilibrium. A xenon lamp with a power of 125 W was employed as a light source for the photocatalytic experiment. The light was channeled through a 1 mm diameter optical fiber (THORLABS) and directed onto the sample cuvette. We conducted the photocatalytic experiment using the full spectrum (UV–vis) of the xenon lamp. During the real-time measurements, we collected absorption data at 1 min intervals for a total of 180 min.

## 3 Results

### 3.1 Powder X-ray Diffraction (PXRD) Analysis

**Figure 2:**
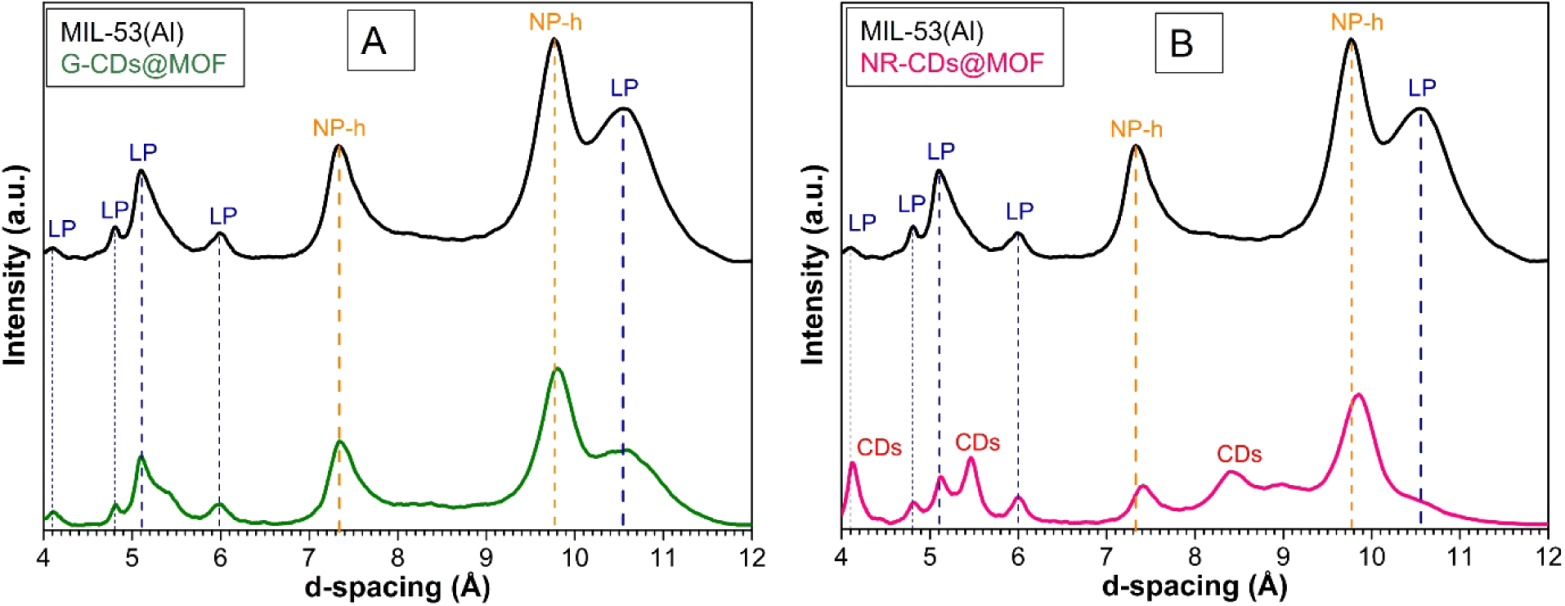
(A) PXRD patterns of pristine MIL-53(Al) and G-CDs@MOF; (B) pristine MIL-53(Al) and NR-CDs@MOF composites. The vertical dashed lines highlight the most stable crystalline phases of MIL-53(Al) in blue for the large pore (LP) and in orange for the hydrated narrow pore (NP-h); additional peaks related to the presence of CDs are labeled accordingly.

PXRD analysis was performed to investigate the crystalline structures of the pristine MIL-53(Al) MOF, G-CDs, and NR-CDs and their corresponding composites. The XRD patterns shown in Figures 2A,B) and Figure S1A,B reveal insightful information regarding the structural phases and the impact of CD integration on the MOF framework. For our convenience, the 2θ scale of the reported PXRD data was converted into *d*-spacing by using the Bragg’s equation to assess the possible structural transformations more clearly.^27^

Furthermore, for broader perspectives, the previously reported structural features of this material are also presented in the form of differently colored vertical dashed lines corresponding to the two possible stable crystalline phases of MIL-53(Al) MOF, known as the anhydrous large pore (LP) phase, where the pores are empty, and the hydrated narrow pore (NP-h) phase, where guest molecules are present inside the cavities of the framework.^27^ In this case, the blue lines represent the LP phase, and the orange lines represent the corresponding NP-h phase, with the thickness of the lines representing the intensity of the peaks, respectively. It is observed that the PXRD pattern of pristine MIL-53(Al) exhibits characteristic peaks corresponding to both the LP and the NP-h phases. The presence of this dual phase reveals the intrinsic flexibility of the MIL-53(Al) framework, which can adapt its pore structure in response to external stimuli or guest molecules.

The PXRD pattern of G-CDs@MOF shows that this composite essentially keeps the LP and NP-h phases of the pristine MIL-53(Al) MOF. However, both a notable reduction in the relative intensity of the main peaks and a change in their shape are observed. On the other hand, as shown in Figures 2B and S1B, the PXRD spectrum of NR-CDs@MOF exhibits some additional peaks that are associated with the characteristics of pristine NR-CDs, in agreement with their integration into the MOF structure. Remarkably, the relative intensity of the peaks related to the LP phase of MIL-53(Al) is significantly reduced, indicating a predominance of the NP-h phase in this composite.

Regarding the pristine CDs, as presented in Figure S1A,B, the PXRD pattern of G-CDs shows a single prominent peak at approximately 2θ = 26°, which is characteristic of the CDs’ graphitic structure.^28^ On the other hand, in the case of NR-CDs, the PXRD evidence additional peaks in addition to the main one at 2θ = 27°, which are mostly related to the presence of NaCl impurities.^26^

### 3.2 Thermogravimetric Analysis and Differential Thermal Analysis

TGA and DTA were conducted to evaluate the thermal stability and decomposition response of MIL-53(Al), green- and neutral-red CDs, and their respective composites.

**Figure 3:**
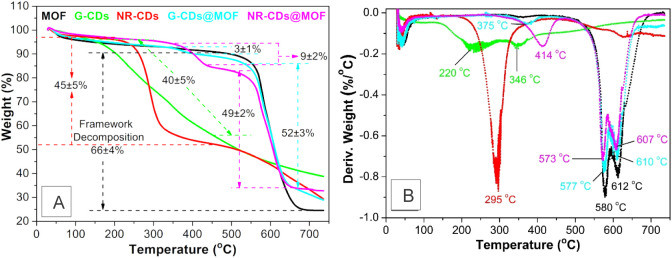
(A and B) TGA and DTA curves of pristine MIL-53(Al), G-CDs and NR-CDs in comparison with the G-CD@MOF and NR-CD@MOF composites.

As depicted in Figure 3A,B, for the pristine MIL-53(Al) MOF, the TGA curve (black) exhibits two weight loss events, as evidenced from the peaks observed in the relative DTA curve. The first weight loss of about 3% occurs below 100 °C and is attributed to the removal of physically adsorbed water and solvent molecules.^3,19^ The second, more significant weight loss of approximately 66 ± 4% occurs in two steps recorded at temperatures of 580 and 612 °C; this weight loss corresponds to the overall decomposition of the MOF framework and the loss of its organic linkers.^3,19^

In the case of the MIL-53(Al) composite with G-CDs, the TGA curve (dark green) shows an additional weight loss of 3 ± 1% around 375 °C. This extra weight loss indicates the presence of a thermally labile component introduced by the incorporation of G-CDs.^29^ Following this, the composite exhibits a major weight loss of 52 ± 3% taking place at high temperature and corresponding to the decomposition of its framework; as for the pristine MIL-53(Al) MOF, this event occurs in two steps at 577 and 610 °C.

For the MIL-53(Al) composite with NR-CDs, compared to the pristine MIL-53(Al) MOF, an additional weight loss of 9 ± 2% is observed at approximately 440 °C. This significant weight loss is indicative of the decomposition of the NR-CDs within the composite. Subsequently, this composite also experiences a major two-step weight loss of 49 ± 2% at 573 and 607 °C.

When it comes to the pristine CDs, the TGA curve of NR-CDs displays a considerable weight loss of 45 ± 5% at around 295 °C, while the G-CDs exhibit a weight loss of 40 ± 5% occurring at the temperature of 220 and 346 °C. In the case of both pristine CDs, we ascribe such a decrease in weight to the decomposition of their structure, possibly occurring through the loss of their surface shell moieties.^30^ Notably, such decomposition occurs at lower temperatures compared to what was recorded for both CDs@MOF composites; the witnessed increased stability suggests a successful incorporation of the nanoparticles inside the MOF’s structure.

Additionally, TGA data allow us to estimate the amount of CDs mass loaded in the framework, considering the decomposition profiles of both CDs and the MOF. To this end, we assume that the additional weight losses observed in the CDs-MOF composite (that are not present in the pristine MOF) are solely due to CDs and that the observed TGA process evolves in the same way as for free CDs. According to this hypothesis, from the observed mass loss of 9 ± 2% in NR-CDs@MOF, we obtain that the amount of NR-CDs is approximately 20 ± 7% of the total mass of the composite. In the case of G-CDs@MOF, from the observed 3 ± 1% mass loss, we determine that the amount of G-CDs is 8 ± 2% of the total mass.

### 3.3 UV–Vis Absorption Spectroscopy

**Figure 4:**
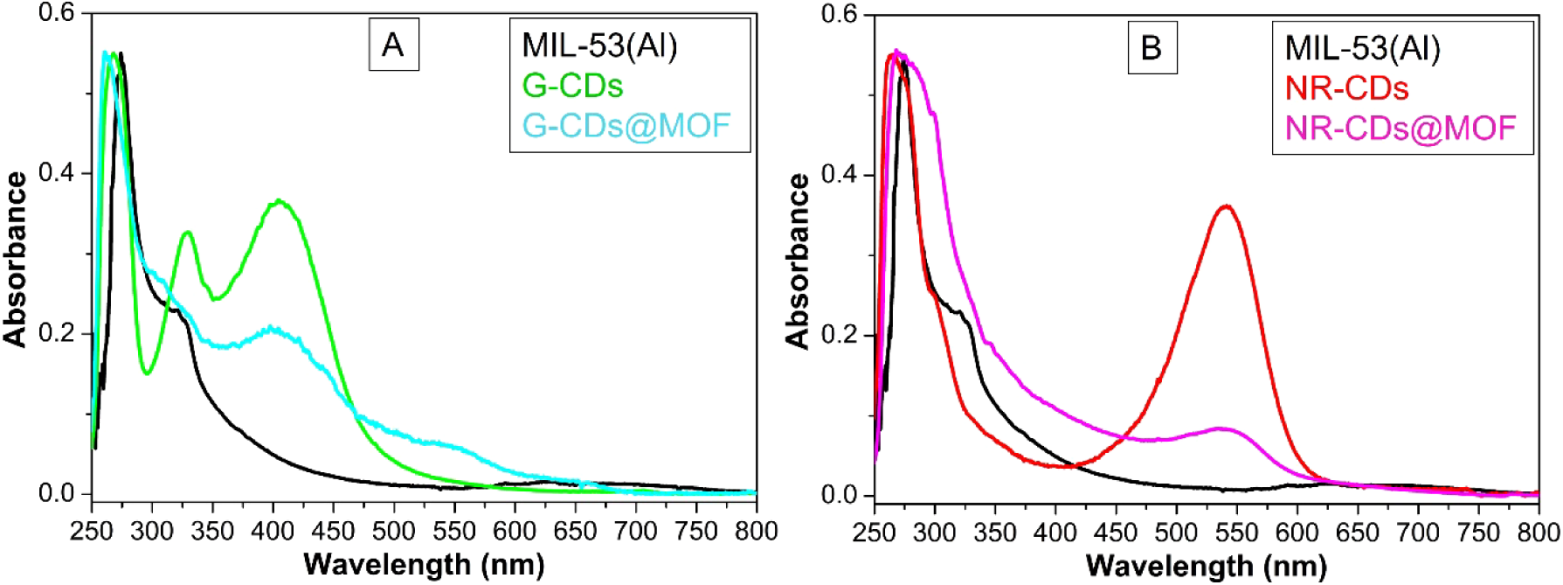
(A) UV–vis absorption spectra of pristine MIL-53(Al), G-CDs, and G-CDs@MOF composites. (B) UV–vis absorption spectra of NR-CDs and NR-CDs@MOF composites in comparison with MIL-53(Al).

UV–vis absorption spectroscopy was employed to investigate and compare the optical properties of the pristine MIL-53(Al) MOF, CDs, and their respective composites in aqueous dispersion. As displayed in Figure 4A, the pristine MIL-53(Al) MOF exhibits two absorption bands in the UV region, peaking at 275 and 325 nm, indicating characteristic electronic transitions.^31^ On the other hand, pristine G-CDs show two sharp absorption bands at wavelengths of 268 and 329 nm in the UV region, in conjunction with a wide and intense band in the visible region centered at 405 nm. Similarly, when G-CDs are incorporated within the MOF, the subsequent composite displays comparable absorption features, with small shifts in the peak’s wavelengths and a considerable reduction in the intensity of these absorption bands. Figure 4A also shows the emergence of a new band at around 550 nm in the G-CDs@MOF composite. Although the origin of this band remains an open question, we can deduce that it arises from interactions between the CDs and the host MOF network, leading to the formation of new electronic states. This hypothesis is consistent with previous works, which have highlighted how the optical properties of CDs are strongly influenced by interactions with the surrounding environment via electron or energy transfer mechanisms.^24−26^ Moreover, as depicted in Figure 4B, pristine NR-CDs present an absorption spectrum with two bands in the UV region at wavelengths of 276 and 300 nm, along with a prominent absorption maximum in the visible region at 541 nm. The NR-CDs composite with MOF retains these absorption features, showing only minor shifts in wavelength and significantly reduced intensity of the absorption bands compared to the pristine NR-CDs. We note that a blue shift of ∼5 nm in the wavelengths is common to both types of CDs when incorporated into the MOF network; this suggests the interaction between the CDs and the host matrix likely influences the electronic properties of the composite.^32^

### 3.4 Time-Resolved Photoluminescence Spectroscopy

To understand the photophysical response of our samples, we recorded the PL spectra of pristine MIL-53(Al) MOF and its composites with G- and NR-CDs, in both solid-state and aqueous dispersion, under UV (305 nm) and visible (440 and 532 nm) excitation.

**Figure 5:**
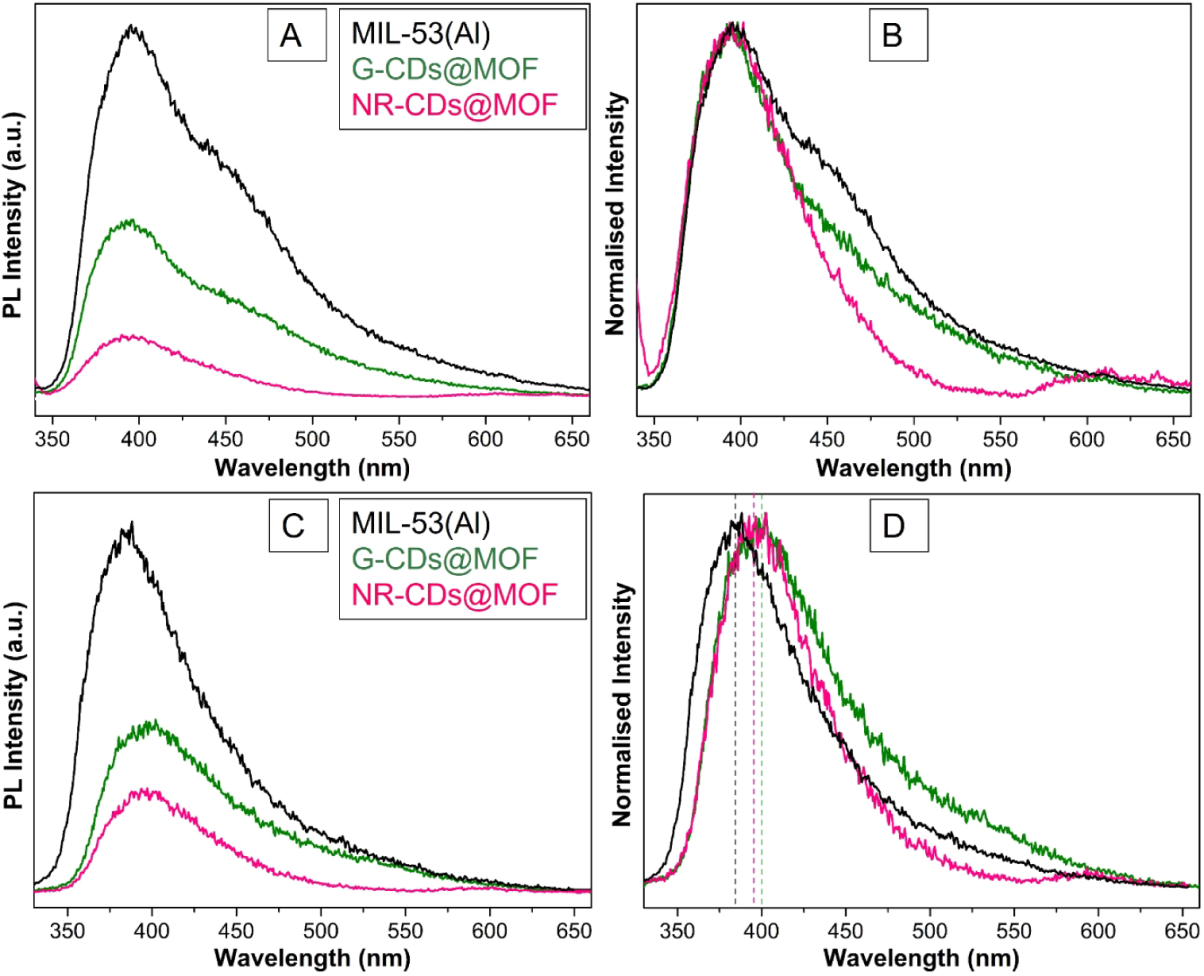
(A) Photoluminescence and (B) normalized photoluminescence emission spectra of MIL-53(Al) and CDs@MOF composites in solid-state form. (C) Photoluminescence and (D) normalized photoluminescence emission spectra of the corresponding samples in aqueous dispersion, excited at λ_excitation_ = 305 nm.

As presented in Figure 5A,B in the solid-state samples, when excited at 305 nm, pristine MIL-53(Al) exhibits two distinct emission bands: a prominent one centered at 395 nm and a second, noticeable as a shoulder at longer wavelengths, centered around 440 nm. The MIL-53(Al) composite with G-CDs also evidence these two bands, but their intensities are markedly reduced compared to the pristine MOF. Instead, when NR-CDs are incorporated, the band at 395 nm is still observed, but with reduced intensity, while the shoulder at 440 nm disappears, and a new, less intense emission emerges with a maximum at around 605 nm.

PL spectra in aqueous dispersion are shown in Figure 5C,D and highlight notable differences compared to solid-state samples. Indeed, the pristine MIL-53(Al) MOF only exhibits a single emission band centered at 385 nm. The composite of MIL-53(Al) with G-CDs gives rise to a single emission band as well, with a peak around 400 nm, red-shifted by 15 nm, and with lower intensity compared to the pristine sample. However, the composite of MIL-53(Al) with NR-CDs shows two emission bands around 395 and 595 nm, as in the case of the photoluminescence spectrum in solid-state samples, but with a blue shift of about 10 nm in the emission maxima of the second band. Once again, the incorporation of NR-CDs reduces the PL intensity compared to the pristine sample in aqueous dispersion.

For completeness, Figure S2 shows the steady-state photoluminescence excitation (PLE) spectra recorded at 400 nm in G- and NR-CDs@MOF composites, at 530 nm in G-CDs@MOF, and at 660 nm in NR-CDs@MOF. The emission at 400 nm, which originates from the MOF network, exhibits a composite PLE profile that extends throughout the UV range, with different peaks, the main ones being around 305, 265, and 210 nm. The PL at 530 nm is related to G-CDs incorporated in the MOF and shows an excitation band centered around 430 nm. The emission at 595 nm (studied at 660 nm to reduce the excitation source scattering contribution) originates from NR-CDs and presents an excitation profile peaked around 570 nm.

Additionally, we measured PL lifetimes of the pristine MIL-53(Al) and its composites with green- and neutral-red-CDs in both solid-state and aqueous dispersion. As reported in Figure S3A,B, the decay curves can be fitted by a single exponential function, from which we get the emission lifetime. We note that the lifetime of the 395 nm emission is poorly influenced by the incorporation of G-CDs and NR-CDs into the MOF network. In the solid state, the pristine MIL-53(Al) exhibits a lifetime τ = 6.0 ± 0.2 ns; the composite of MIL-53(Al) with G-CDs has τ = 5.5 ± 0.2 ns; and the NR-CDs@MIL-53(Al) composite has a slightly shorter lifetime τ = 4.9 ± 0.2 ns. In aqueous dispersion, the measured lifetimes are τ = 5.5 ± 0.2 ns in pristine MIL-53(Al) and G-CDs@MIL-53(Al) composite, while the NR-CDs@MIL-53(Al) composite has *t* = 6.5 ± 0.2 ns. The reported errors correspond to the standard deviation of the best-fitting parameters obtained from the least-squares regression.

**Figure 6:**
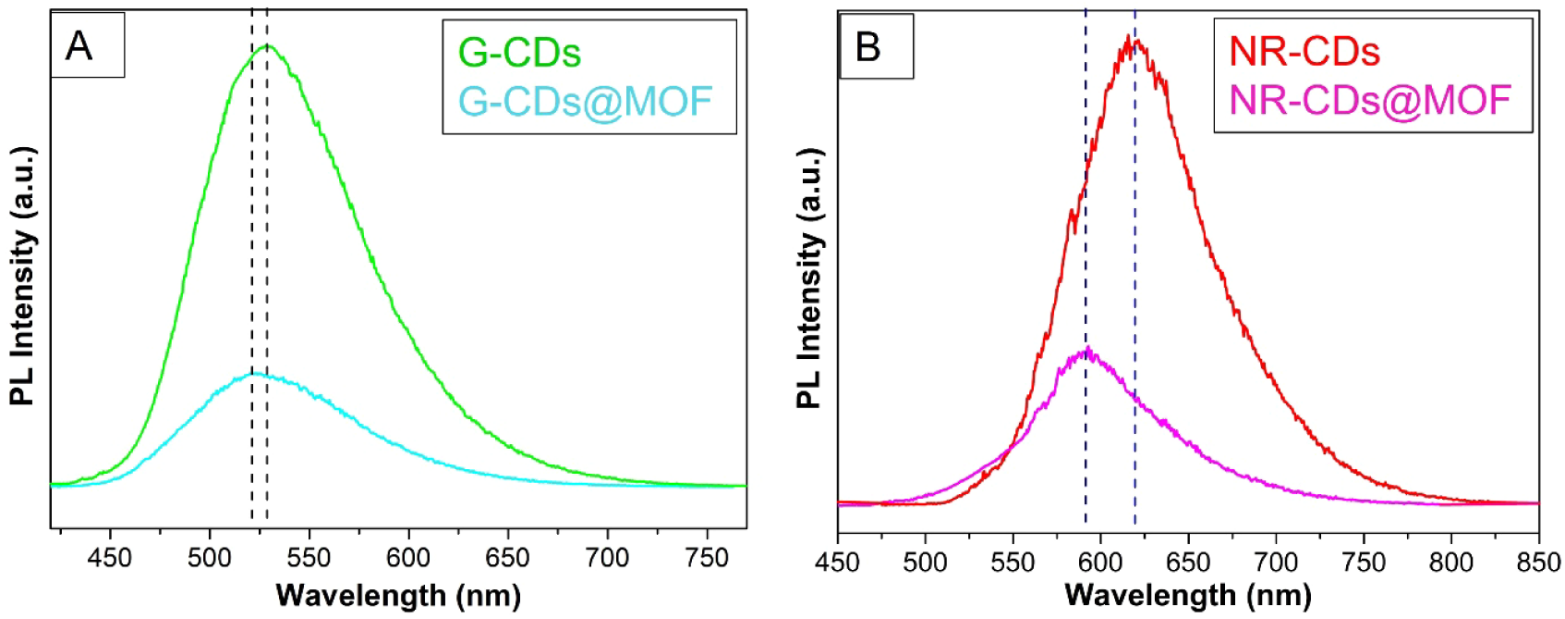
(A) Emission spectra of pristine G-CDs and G-CD@MOF, excited at λ_excitation_ = 440 nm. (B) Emission spectra of pristine NR-CDs and NR-CD@MOF, excited at λ_excitation_ = 532 nm.

To further explore the PL response of these CDs@MOF composites, we also performed PL spectra by exciting in the visible region. As depicted in Figure 6A, when pristine G-CDs are excited at 440 nm, an emission band is observed around 528 nm, whereas the G-CDs@MIL-53(Al) composite displays a slightly blue-shifted and lower-intensity emission band at 521 nm. As reported in Figure S4, the lifetime of these bands is τ = 7.2 ± 0.2 ns for pristine G-CDs and G-CDs@MIL-53 (Al) composite, respectively. Figure 5B reports the spectra of pristine NR-CDs and the NR-CDs@MIL-53(Al) composite under excitation at 532 nm. In the pristine sample, an emission band is observed around 620 nm, while the composite exhibits a band centered at 590 nm with lower intensity. The corresponding decay curves shown in Figure S4 indicate that the PL in NR-CDs has a lifetime τ = 5.3 ± 0.2 ns, while in NR-CDs@MIL-53(Al) the measured value τ = 1.3 ± 0.2 ns is comparable with the decay time of the excitation laser pulse and, therefore, represents an upper limit of the true lifetime.

### 3.5 Photocatalytic Performance Evaluation

The photocatalytic performance of the CDs@MOF composites was thoroughly tested and compared with those of the pristine CDs and MIL-53(Al) MOF, using Rhodamine B (RhB) as a model dye. Initial evaluation of the photodegradation response of RhB was carried out without any catalyst across different pH levels (4, 7, and 9). As shown in Figure S5A,B,C, RhB remained stable over a duration of 180 min at all tested pH levels under irradiation.

However, significant differences were observed when various catalysts were introduced. As shown in Figures S6A,D and 7A–C, after 180 min at pH 9, the bare G- and NR-CDs achieve 15% and 16% degradation rates, the pristine MIL-53(Al) MOF shows a 25% degradation of RhB, while the G-CD@MOF and NR-CD@MOF composites reach degradation rates of 27% and 35%, respectively. As depicted in Figures S6B,E and 7D–F, at pH 7, the efficiency of the RhB photodegradation remains almost the same for bare CDs; it is 37% in the pristine MIL-53(Al) MOF, 44% in G-CD@MIL-53(Al), and 52% in NR-CD@MIL-53(Al). Finally, as shown in Figures S6C,F and 7G,I, at pH 4, G-CDs and NR-CDs accomplish the degradation efficiency of 19% and 20%, respectively. Pristine MIL-53(Al) MOF reaches a 51% degradation, G-CD@MIL-53(Al) achieves 56%, and the NR-CD@MIL-53(Al) composite evidences an outstanding 90% photodegradation of RhB. These results demonstrate the improved photocatalytic efficiency in the CDs@MOF composites, predominantly for the NR-CD@MIL-53(Al), which meaningfully outperforms bare CDs and the pristine MIL-53(Al) MOF under all tested pH values.

**Figure 7:**
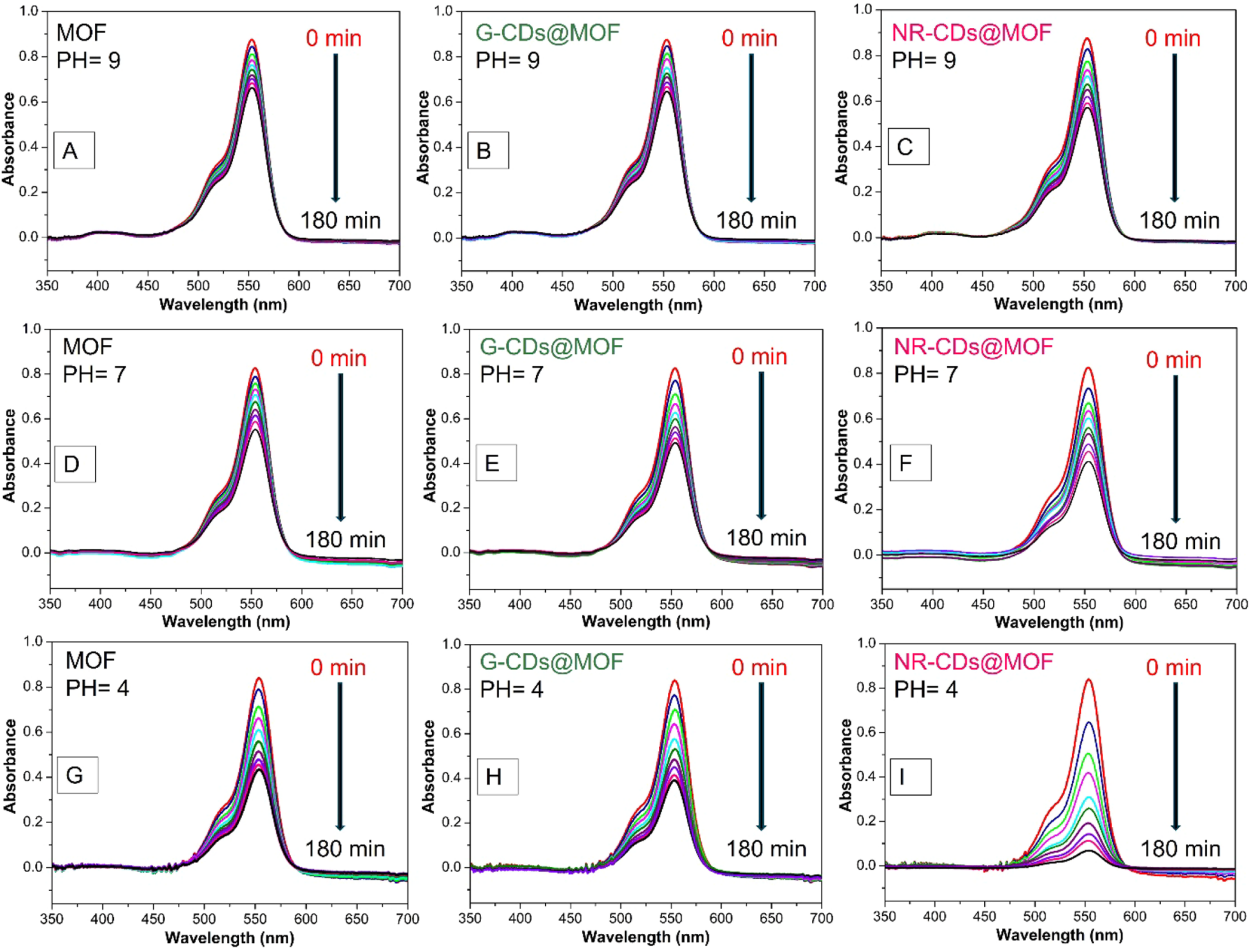
(A–I) Photocatalytic activity of CDs@MIL-53(Al) composites in comparison with pristine MIL-53(Al) MOF for RhB photodegradation at different pH levels.

**Figure 8:**
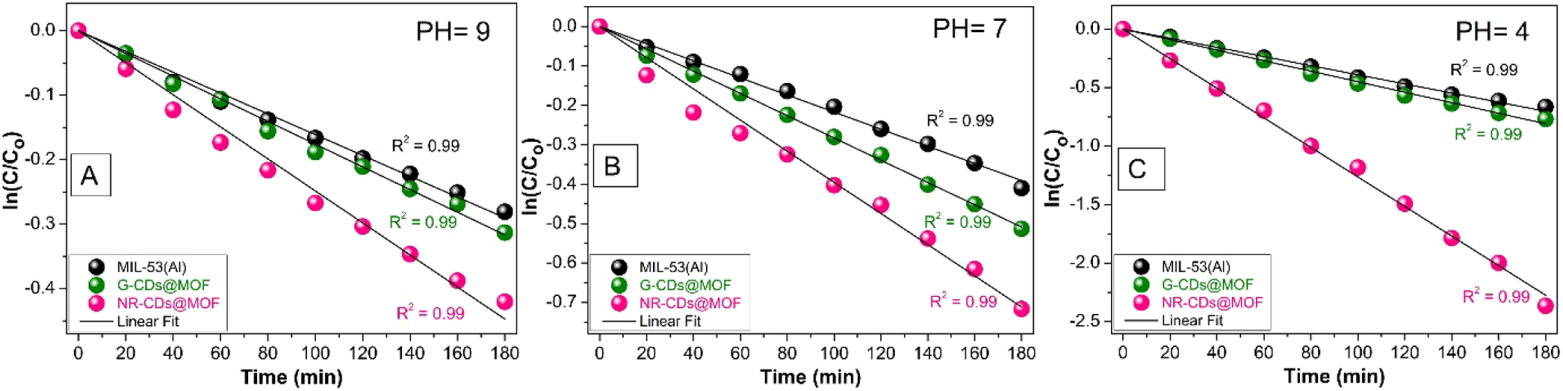
(A–C) ln(C/C_0_) vs time curves at different pH levels. All data points are represented with colored spheres, and the straight black lines represent the linear fit of the data points.

Moreover, as displayed in Figure 8A–C, the reaction kinetics of the photocatalytic process are examined, disclosing that the photodegradation of RhB follows a pseudo-first-order reaction kinetics, as verified by the linear fitting of all the photodegradation curves. The photodegradation rate constants at different pH levels are also calculated to better point out the efficiency of the different catalysts.

It is observed that, at pH 7, the rate constant for the pristine MIL-53(Al) MOF is measured to be (2.19 ± 0.03) × 10^–3^ min^–1^. The G-CD@MOF composite exhibits an improved rate constant of (2.78 ± 0.03) × 10^–3^ min^–1^, while the NR-CD@MOF composite displays the highest rate constant of (3.67 ± 0.09) × 10^–3^ min^–1^, respectively. These values highlight that the integration of CDs enhances the photocatalytic performance of the MOF, with the NR-CD@MIL-53(Al) composite performing better than both the pristine MIL-53(Al) and the G-CD@MIL-53(Al) composite.

In addition, at pH 9, the rate constants are lower, reflecting a decrease in the photocatalytic performance under more basic pH conditions. The pristine MIL-53(Al) MOF has a rate constant of (1.53 ± 0.03) × 10^–3^ min^–1^, the G-CD@MIL-53(Al) composite exhibited a slight enhancement with a rate constant of (1.70 ± 0.03) × 10^–3^ min^–1^, and the NR-CD@MIL-53(Al) composite once again displays a better performance with a rate constant of (2.32 ± 0.07) × 10^–3^ min^–1^.

However, the most considerable performance is observed at pH 4, where the photocatalytic activity is meaningfully enhanced. The pristine MIL-53(Al) has a rate constant of (3.82 ± 0.06) × 10^–3^ min^–1^, the G-CD@MOF composite improves to (4.44 ± 0.06) × 10^–3^ min^–1^, and intriguingly, the NR-CD@MOF composite presents an outstanding rate constant of (12.92 ± 0.14) × 10^–3^ min^–1^. These findings highlight the remarkable photocatalytic performance of the NR-CD@MOF composite, especially in acidic environments, and are ascribed to the incorporation of CDs that improve the light absorption as well as the charge separation in the composite.^33^

## 4 Discussion

The PXRD pattern of MIL-53(Al) exhibits distinctive peaks that are indicative of the NP-h phase as well as the LP phase, demonstrating the inherent flexibility of the framework.^27^ This dual phase suggests that MIL-53(Al) can dynamically transform its structure in response to external stimuli or guest molecules in particular; in the case of the pristine MOF, the effect of surrounding atmospheric molecules is predominant, since the measurements were performed in an open environment. Additionally, the integration of CDs into the MIL-53(Al) framework not only influences the structural properties of the composite, as evidenced by PXRD patterns, but also impacts the thermal stability and decomposition response of its components, as analyzed by TGA analysis.

The incorporation of G-CDs transforms the crystallinity of the MIL-53(Al) structure, introducing partial amorphization or structural defects without changing the porosity of the MOF. As only slight changes in the shape of PXRD peaks and their relative intensity are observed, we hypothesize that, for the G-CDs@MIL-53(Al) composite, G-CDs reside only on the surface of the MOF and develop weak interactions with the framework. Nonetheless, this interaction provides enhanced thermal stability to G-CDs@MOF, as indicated in the TGA analysis by the weight loss associated with this decomposition: this occurs at a temperature of 375 °C instead of 220 °C, as in the case of bare G-CDs. These results indicate the existence of a thermally labile component of CDs weakly coupled to the MOF.^34^

In contrast, NR-CDs induce more pronounced structural changes, as evidenced by the significant reduction in the relative intensity of some PXRD peaks, especially the characteristic LP at the *d*-spacing of 10.54 and 5.10 Å. The comparison between the pristine MOF and the composite NR-CDs@MOF also highlights the different positions of some peaks: the one at 4.10 Å in the MOF is shifted to 4.14 Å, while the characteristic peaks of the NP-h phase, observed at 9.77 and 7.32 Å in the MOF, are shifted to 9.85 and 7.42 Å, respectively. Although PXRD analysis does not provide direct evidence of the exact positioning of the CDs in the framework, it can be hypothesized that the NR-CDs do not simply surround the surface of the MOF but that a significant fraction fits well inside the MOF by the defect cavities. As a result, strong hydrogen bonds and electrostatic interactions are established,^36^ which stabilize the NP-h phase of the MOF. Previous studies have proven that the presence of structural defects in MOFs, such as missing linkers, metal vacancies, and node distortions, can notably influence their physicochemical response. These imperfections not only enhance properties such as the diffusion of guest molecules and catalytic efficiency but also create large accessible cavities within the framework. Furthermore, these cavities can also accommodate guest molecules beyond the conventional pore sizes of the pristine MOFs. In the NR-CD@MOF system, this hypothesis provides a probable basis for the partial inclusion of large-sized NR-CDs within the MOF pores. This presence likely contributes to their enhanced thermal stability, as reflected in our TGA analysis, where the embedded NR-CDs are protected from rapid thermal dissociation, either via spatial confinement or electronic interactions with the MOF scaffold.^37,38^

The photophysical characteristics and excited-state dynamics of the pristine MIL-53(Al) MOF and its composites with CDs, both in solid-state form and in aqueous dispersion, were better understood by performing PL measurements.

The PL spectra detected under UV excitation are mainly from the MIL-53(Al) structure. In fact, different studies have proposed that, in pristine MIL-53(Al), the emissions at 395 and 440 nm correspond to intraligand transitions and ligand-to-metal charge transfer, respectively.^21,39^ The incorporation of CDs can give rise to several processes that influence the PL bands. On the one hand, they favor nonradiative pathways, such as the charge transfer from MOF to CD, thus reducing the PL intensity. As highlighted in Figure S3A,B, this quenching process does not significantly alter the PL lifetime recorded at 395 nm. We infer that the radiative decay rate (*k*_*r*_) that governs the intrinsic lifetime τ of intraligand transition is largely determined by its inherent electronic structure and is not affected by the interaction with CDs. The intensity changes are driven by ultrafast nonradiative (*k*_nr_) processes, which limit the population of the excited state before the emission is triggered; therefore, they do not compete with radiative decay in governing the lifetime. On the other hand, CDs can alter the electronic environment of the emissive states and modify the transition energy, as evidenced by the PL red shift in aqueous dispersion. CDs can also act as energy acceptors, emitting in lower-energy states.^23−26^ This is consistent with the observation of PL around 605 nm in the NR-CDs@MOF composite, where charge transfer complexes form between the absorbing MOF and the emitting NR-CDs. Furthermore, these processes could overwhelm the charge transfer from the ligand to the metal, favoring the quenching of the related PL at 440 nm.

Regarding visible excitation, the emission response is mainly dictated by CDs and their interactions with the MOFs. It is known that the G-CDs and NR-CDs emission is strongly coupled to the local environment through their surface^25,26^ and can thus be considerably influenced when incorporated into the MOF. We infer that the MOF can act as a rigid scaffold, constraining the CDs and modifying their surface states, resulting in variations in the PL spectra. This may explain the observed blue shifts that may be associated with more polar environments in composites, which stabilize higher-energy excited states.^25,26,40^

The UV–vis absorption results for the pristine MIL-53(Al) MOF, CDs, and their composites reveal significant insights into the optical properties and interactions within these materials. Two distinct absorption bands of pristine MIL-53(Al) correspond to the π–π* transitions in the organic linker and the charge transfer interactions between the aluminum centers and the organic framework.^21,41,42^ Pristine CDs also show absorption bands in the UV region, along with a broad and intense band in the visible region. These absorption bands likely arise due to π–π* or *n*–π* transitions or, more likely, due to surface states or defect states within the CDs.^26,34,35^ These states can arise from the functional groups on the surface of CDs, indicative of their unique electronic structure. Moreover, upon integration of CDs with the MOF, the composite displayed absorption features that were similar to the pristine nanostructures but with slight shifts in the wavelengths of the absorption bands and a significant reduction in peak intensity. These changes suggest a successful interaction between CDs and the MOF, which likely modifies the local electronic environment of the composite. These shifts in absorption bands and the lower intensities point to a possible electronic coupling between CDs and MIL-53(Al) framework.^25,36,40,41^ The interaction between the CDs and the MOF matrix leads to modifications in the electronic structure,^36,40,42^ which can enhance the material’s potential for applications in photocatalysis and other optoelectronic fields. These findings suggest that the composites can be fine-tuned for specific applications by leveraging the unique optical and electronic properties of both the MOF and the CDs.

The photocatalytic mechanism of bare CDs, MIL-53(Al), and CDs@MOF composites can be elucidated by considering the roles of each component and their interactions during the photodegradation of RhB. The relatively lower photocatalytic efficiency of bare CDs could be linked to the following factors. As the CDs possess various surface functional groups, their surface area and the availability of active sites are relatively limited compared to more structured porous materials like MOFs. This limitation may reduce the interaction between the catalyst and the target molecules, leading to a lower photocatalytic efficiency. Moreover, CDs are highly sensitive to environmental pH, which influences their surface charge and electronic structure. At certain pH levels, the catalytic activity of CDs might be hampered due to reduced interaction with RhB or deactivation of functional groups.

**Figure 9:**
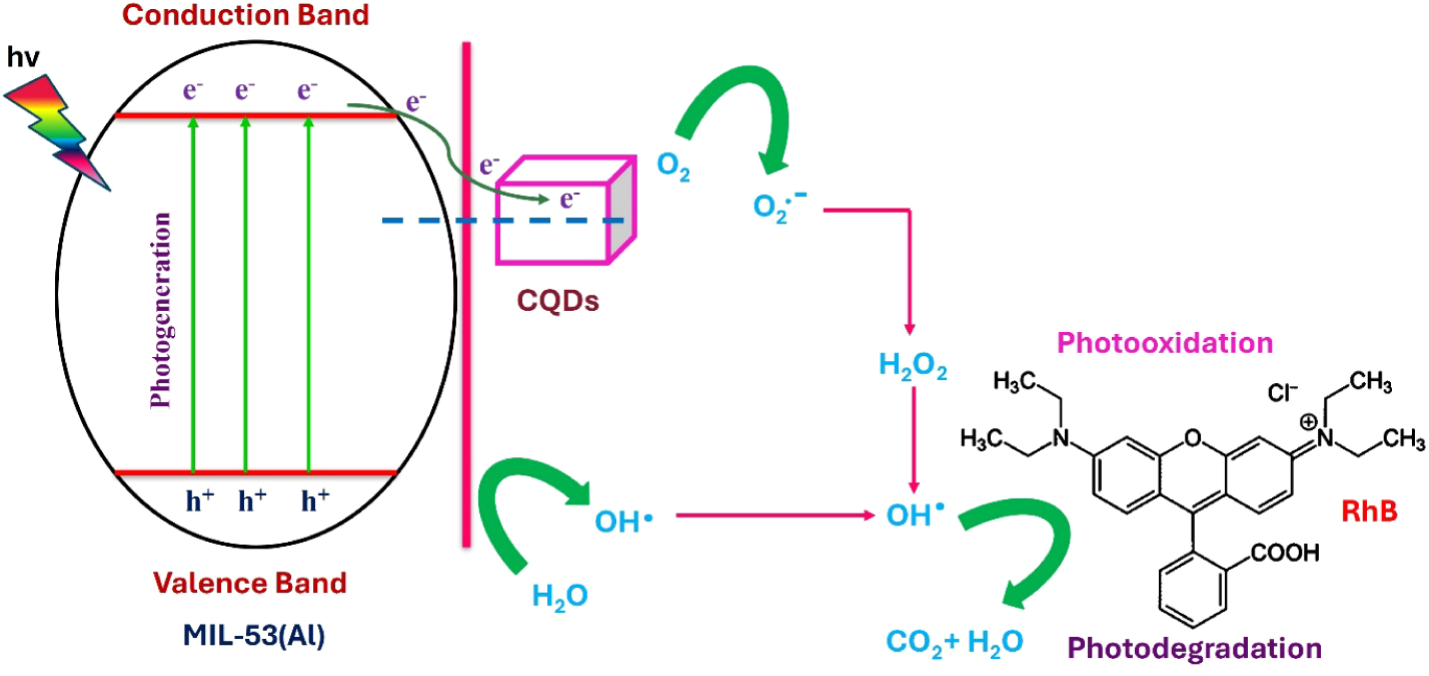
Photocatalytic mechanism of Rhodamine B (RhB) photodegradation.

As depicted in the mechanistic Figure 9, the incorporation of G-CDs and NR-CDs into the MIL-53(Al) MOF matrix enhances photocatalytic efficiency through several key mechanisms. (i) Upon illumination with UV–visible light, the MIL-53(Al) MOF and CDs absorb photons, exciting electrons from the valence band (VB) to the conduction band (CB), generating electron–hole pairs. (ii) The pristine MIL-53(Al) MOF predominantly absorbs in the UV region, while CDs extend the absorption into the visible region, broadening the spectrum of light that can be utilized for photocatalysis.^21−23^ (iii) In the pristine MIL-53(Al), photoexcited electrons and holes can recombine quickly, reducing photocatalytic efficiency.^21,43^ (iv) The integration of CDs enhances charge separation due to their ability to trap electrons, reducing the recombination rate. The heterojunction formed between the CDs and the MOF facilitates the transfer of electrons from the MOF to the CDs.^21−23,44,45^ (v) The photoexcited electrons in the CDs@MOF composites can reduce molecular oxygen (O_2_) to form superoxide radicals (•O_2_^–^). Simultaneously, the holes in the VB can oxidize water (H_2_O) or hydroxide ions (OH^–^) to produce hydroxyl radicals (•OH). These reactive oxygen species (ROS) are highly reactive and can degrade RhB molecules through oxidative reactions.^45,46^

At acidic pH (pH 4), the availability of H^+^ ions can facilitate the formation of hydroxyl radicals (•OH) from water, enhancing the degradation of RhB. The NR-CDs@MOF composite, with its superior charge separation and reactive oxygen species (ROS) generation capabilities, shows remarkable photocatalytic performance under acidic conditions. At neutral pH (pH 7), the balance of H^+^ and OH^–^ ions supports effective reactive oxygen species (ROS) generation, resulting in moderate photocatalytic activity. At basic pH (pH 9), photocatalytic activity decreases due to the reduced availability of H^+^ ions, which are crucial for •OH radical formation. However, the NR-CDs@MOF composite still performs better than the pristine MOF and G-CD@MOF composites, indicating its robust photocatalytic properties.

The modifications in the electronic structure of the MOF after the integration of CDs facilitate better light absorption and improve the generation of electron–hole pairs under illumination. CDs often contain surface functional groups and defects that can act as active sites for photocatalytic reactions. These functional groups can facilitate the adsorption of RhB molecules and enhance the interactions between the catalyst and the substrate. The heterojunction formed between CDs and the MIL-53(Al) framework is decisive for effective charge separation. Moreover, CDs can act as electron sinks, preventing the recombination of photoexcited electrons and holes.^45−47^ This leads to a higher availability of charge carriers for reactive oxygen species (ROS) generation.

The photocatalytic reaction mechanism can be further summarized and understood by the following set of equations: (i) Absorption of photon energy by the MIL-53(Al) MOF and CDs leads to the excitation of electrons from the valence band (VB) to the conduction band (CB).


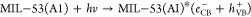

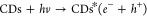


(ii) Transfer of these photoexcited electrons from MOF to CDs, enabling charge separation and reducing electron–hole pair recombination.
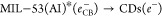


(iii) Generation of reactive oxygen species (ROS) via the reduction of O_3_ to •O_2_^–^ by the electrons and oxidation of H_2_O or OH^–^ to •OH by the holes.
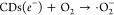

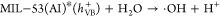

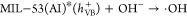


(iv) Degradation of RhB molecules by the reactive oxygen species (ROS), resulting in the degradation of the dye into smaller, harmless products.^45^
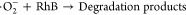

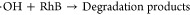


Then, the generation of reactive oxygen species (ROS), such as •O_2_^–^ and •OH, is central to the photocatalytic degradation of RhB.^44,45^ We acknowledge that the formation of ROS could be tested through radical trapping experiments, as demonstrated in^48^ by using different systems as fluorescence probes to quantify the reactive species generated under nonbiological and biologically relevant conditions. In the present work, we assume enhanced reactive oxygen species (ROS) production in the CDs@MOF composites, especially in the NR-CD@MOF, that is attributed to the better charge separation and extended light absorption capabilities of the composite. Moreover, the observed pseudo-first-order reaction kinetics and the rate constants provide quantitative evidence of the improved photocatalytic performance of the CDs@MOF composites. The significantly higher rate constants for the NR-CD@MOF composite, particularly at acidic pH, highlight the effectiveness of the carbon dots in enhancing the photocatalytic activity.

## 5 Conclusions

In conclusion, this study successfully synthesizes and characterizes MIL-53(Al) MOF composites with green- and neutral-red-CDs for photocatalytic applications. It is observed that the CDs integration not only modifies the structural properties of MIL-53(Al) but also influences its optical properties, electronic structure, and photocatalytic performance. Green-CDs induce subtle changes, while neutral-red-CDs cause significant structural alterations. Consequently, the electronic environment of the framework was modified by establishing strong hydrogen bonding and electrostatic interactions. The integration of CDs into the MOF framework significantly enhances the photocatalytic performance, as evidenced by the improved degradation rates of Rhodamine B (RhB) dye under UV–vis light irradiation. The synergistic effects of CDs are attributed to their ability to improve charge separation and extend light absorption, resulting in an enhancement of the photocatalytic performance of the composites. Moreover, structural and optical characterizations confirmed the successful incorporation of carbon dots into the MOF matrix with minimal alteration to the fundamental properties of the MOF structure. These comprehensive findings suggest that CDs@MOF composites hold great promise for tailored applications in catalysis and environmental remediation, with further research needed to optimize their design and understand the underlying mechanisms.

## Supporting Information Available

PXRD patterns of bare CDs and CDs@MOF in comparison with pristine MOF, PLE spectra of CDs@MOF composites, PL decay curves of CDs@MOF in comparison with pristine MOF and relative exponential fit curves, PL decay curves of CDs@MOF in comparison with the bare G-CDs and NR-CDs, photodegradation of RhB without catalysts at different pH levels, and additionally, photodegradation of RhB at different pH levels with the bare CDs as catalysts (PDF)
