# Supplementary material for: Engineering MIL-53(Al) MOF with Carbon Dots for Synergistic Photocatalysis Applications in Organic Dye Degradation
Source: ACS Omega. 2025 Apr 30;10(18):18527–38. doi: 10.1021/acsomega.4c11113 (PMC12079226; doi:10.1021/acsomega.4c11113)
Supplement: Supplementary file 1 — ao4c11113_si_001.pdf [file ao4c11113_si_001.pdf]

Supporting Information

Engineering MIL-53(Al) MOF with Carbon Dots for Synergistic Photocatalysis Applications in Organic-dye Degradation

Tanzeel Ul Rehman[1] *, Simonpietro Agnello[1], Franco Mario Gelardi[1], Antonino Madonia[1], Alice Sciortino[1], Martina Maria Calvino[1], Giuseppe Lazzara[1], Gianluca Minervini[2], Annamaria Panniello[2], Gianpiero Buscarino[1], Marco Cannas[1]

1) Dipartimento di Fisica e Chimica Emilio Segrè, Università degli Studi di Palermo, 90123 Palermo, Italy
2) Institute for Chemical and Physical Processes Bari Division, Italian National Research Council, 70126 Bari, Italy

* e-mail: tanzeelul.rehman@unipa.it

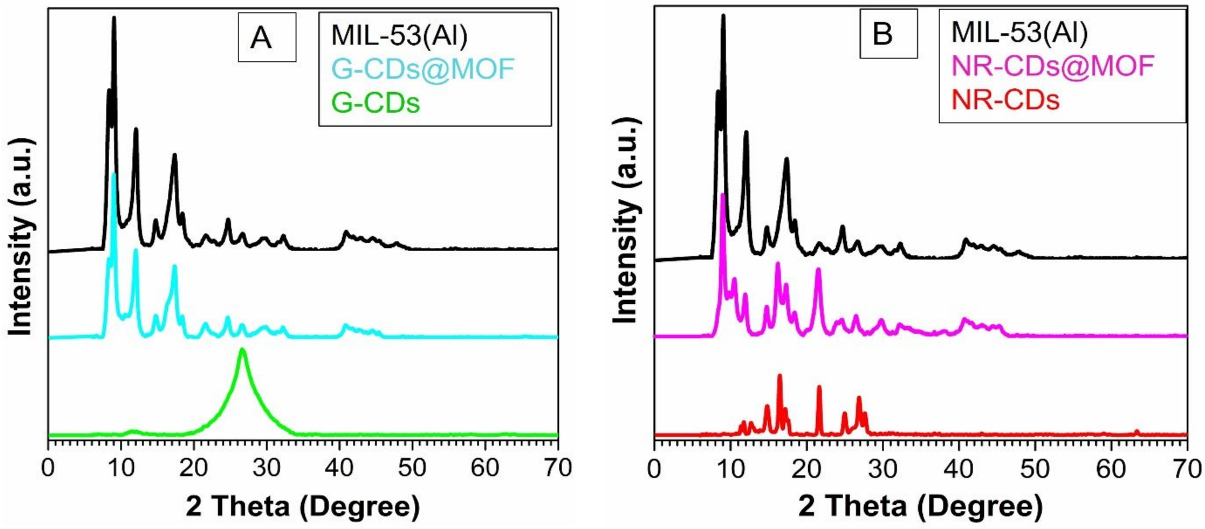

Figure S1: (A) PXRD patterns of pristine MIL-53(Al) MOF, Green carbon dots (CDs) and G-CDs@MOF composite. (B) PXRD patterns of pristine Neutral-Red carbon dots (NR-CDs), and NR-CDs@MOF composites in comparison with pristine MIL-53(Al) MOF.

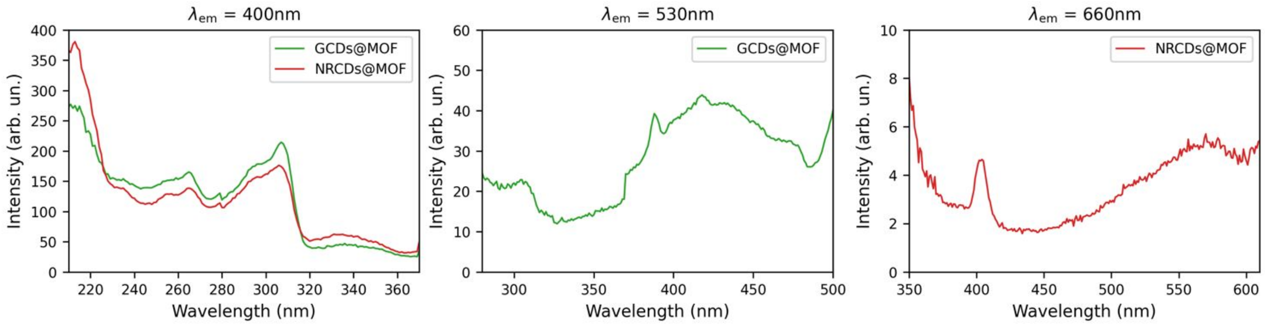

Figure S2: Photoluminescence excitation spectra of G-CDs@MOF and NR-CDs@MOF composites in aqueous dispersion, recorded at 400 nm, 530 nm and 660 nm. The reported profiles are not corrected for the excitation source spectrum and the monochromator response; narrow peaks, for example the one at 390 nm in G-CDs@MOF and the one at 400 nm in NR-CDs@MOF, are instrumental artefacts.

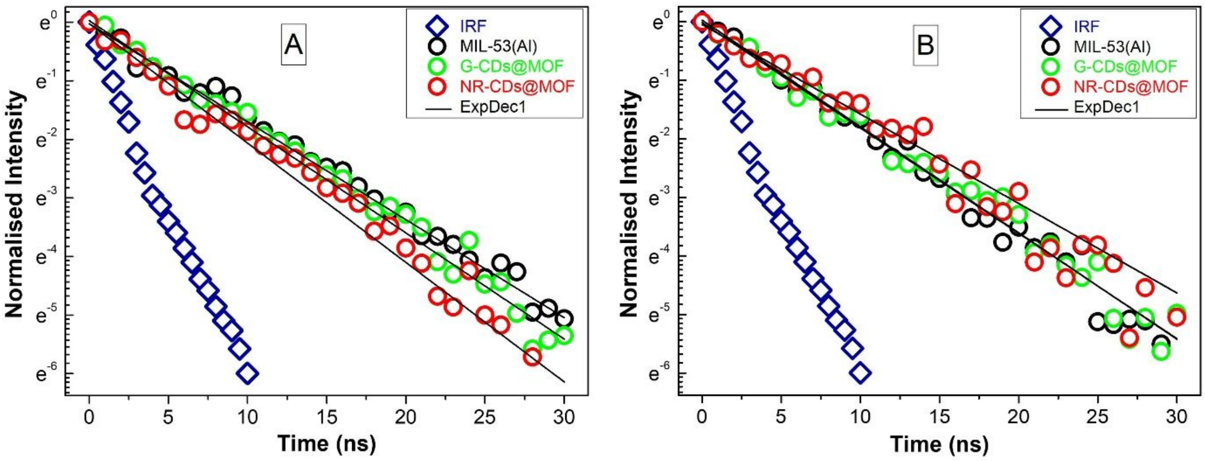

Figure S3: (A) Photoluminescence decay curves of MIL-53(Al) and CDs@MOF composites in solid-state form, monitored at emission wavelength of 395 nm under excitation at 305 nm. (B) Photoluminescence decay curves of MIL-53(Al) and CDs@MOF composites in aqueous solution, recorded at 395 nm and excited at 305 nm. Solid lines are representing the single exponential decay fitting of the decay curves. Blue diamond shapes are representing the instrument response function (IRF) profile.

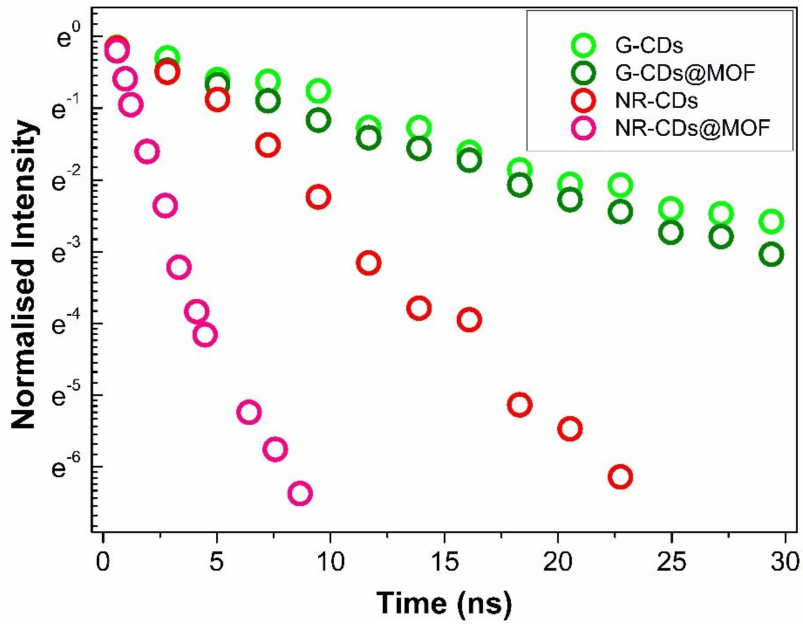

Figure S4: Photoluminescence decay curves of pristine green carbon dots and the green-CD@MOF composite, monitored at 525 nm under excitation at 440 nm, and of pristine neutral-red carbon dots and the neutral-red-CD@MOF composites, recorded at 610 nm and excited at 532 nm.

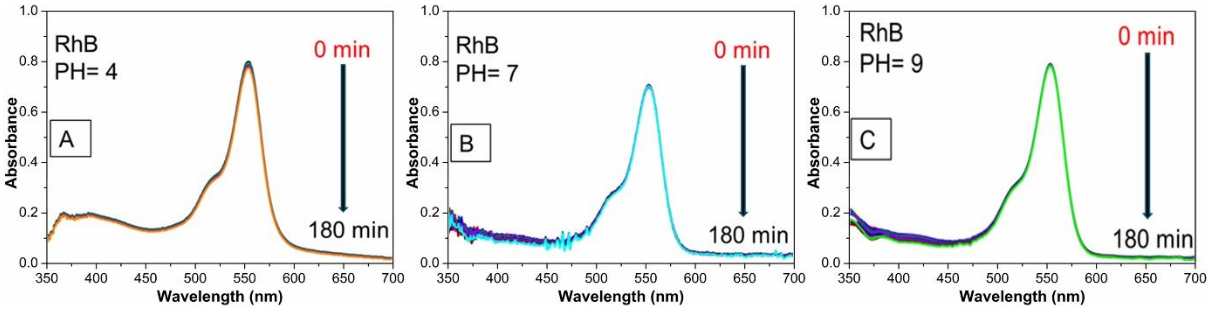

Figure S5: (A-C) Photodegradation of Rhodamine B (RhB) for 180 min without any catalyst at different pH levels (4, 7, and 9).

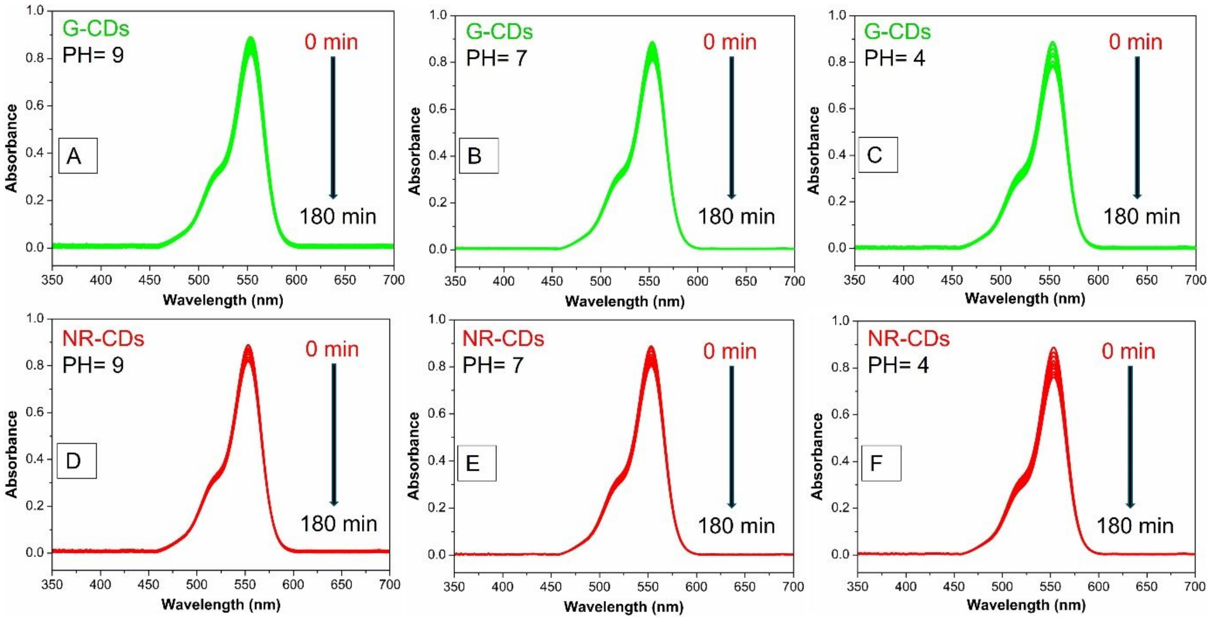

Figure S6: (A-C) Photodegradation of Rhodamine B (RhB) for 180 min with bare G-CDs as a catalyst. (D-F) with bare NR-CDs as a catalyst at different pH levels (4, 7, and 9).
